# Climate Change at Northern Latitudes: Rising Atmospheric Humidity Decreases Transpiration, N-Uptake and Growth Rate of Hybrid Aspen

**DOI:** 10.1371/journal.pone.0042648

**Published:** 2012-08-06

**Authors:** Arvo Tullus, Priit Kupper, Arne Sellin, Leopold Parts, Jaak Sõber, Tea Tullus, Krista Lõhmus, Anu Sõber, Hardi Tullus

**Affiliations:** 1 Department of Silviculture, Institute of Forestry and Rural Engineering, Estonian University of Life Sciences, Tartu, Estonia; 2 Department of Botany, Institute of Ecology and Earth Sciences, University of Tartu, Tartu, Estonia; 3 Wellcome Trust Sanger Institute, Wellcome Trust Genome Campus, Hinxton, Cambridge, United Kingdom; 4 Donnelly Centre for Cellular and Biomolecular Research, University of Toronto, Toronto, Ontario, Canada; University of Nottingham, United Kingdom

## Abstract

At northern latitudes a rise in atmospheric humidity and precipitation is predicted as a consequence of global climate change. We studied several growth and functional traits of hybrid aspen (*Populus tremula* L.×*P. tremuloides* Michx.) in response to elevated atmospheric humidity (on average 7% over the ambient level) in a free air experimental facility during three growing seasons (2008–2010) in Estonia, which represents northern temperate climate (boreo-nemoral zone). Data were collected from three humidified (H) and three control (C) plots, and analysed using nested linear models. Elevated air humidity significantly reduced height, stem diameter and stem volume increments and transpiration of the trees whereas these effects remained highly significant also after considering the side effects from soil-related confounders within the 2.7 ha study area. Tree leaves were smaller, lighter and had lower leaf mass per area (LMA) in H plots. The magnitude and significance of the humidity treatment effect – inhibition of above-ground growth rate – was more pronounced in larger trees. The lower growth rate in the humidified plots can be partly explained by a decrease in transpiration-driven mass flow of NO_3_^−^ in soil, resulting in a significant reduction in the measured uptake of N to foliage in the H plots. The results suggest that the potential growth improvement of fast-growing trees like aspens, due to increasing temperature and atmospheric CO_2_ concentration, might be smaller than expected at high latitudes if a rise in atmospheric humidity simultaneously takes place.

## Introduction

Global warming will be accompanied by a change in atmospheric water vapour content and precipitation rate, although there will be pronounced regional differences in the magnitude and direction of these events [1]. This has been validated by modern climate models [2], [3], [4], as well as by studies on previous global warming periods in the Earth's history [5]. At northern latitudes both precipitation and atmospheric water content will likely increase substantially [6], [7]. In boreal and nemoral regions the predicted rise in annual precipitation varies from 5 to 30%, although it could even be as high as 40%, the change being higher regarding winter precipitation [6], [8]. However, ecosystem responses to changing precipitation differ largely not only among sites, but also yearly at a given site [9], and interactive effects of multiple global change factors on ecosystem processes are complex [10].

Water vapour acts as one of the most important greenhouse gases [3], [11], [12], which absorbs infrared radiation and thus increases air temperature, and consequently, also atmospheric water-holding capacity according to the Clausius-Clapeyron relation [4]. Such a process is known as a water vapour feedback and its potential role in future climate warming is considered to be substantial [11], [13].

Higher relative air humidity (RH) reduces the water vapour pressure difference between the plant leaf interior and the surrounding atmosphere (VPD_L_), a primary driving force of transpiration. The plant transpiration response to VPD_L_ depends on stomatal conductance and parameters regulating it, e.g. soil water potential [14], [15], [16]. We could expect enhanced biomass production of the trees under elevated atmospheric humidity, as decreasing VPD_L_ allows higher stomatal conductance, observed in both broad-leaved and coniferous species [14], [17], [18], [19], [20]. Moreover, reduced transpiration rate under low VPD_L_ can increase water potential and turgor pressure of plant cells, which promote cell expansion, i.e. plant growth [21], [22]. At the same time the responses to combined changes in atmospheric and soil moisture vary remarkably among species [23].

Anatomical and chemical characteristics of tree leaves - the main photosynthetic organs where gas exchange between tree and atmosphere occurs - are widely used to describe nutrition and photosynthetic capacity of the tree in the given environment. The results concerning changes in leaf characteristics at high RH are rather contradictory, reporting both increase [24], [25], [26] and decrease [27], [28] in leaf area and leaf expansion rate at high RH.

Some studies have indicated that trees respond to changes in atmospheric conditions with improved water-use efficiency (WUE; i.e. biomass increment or carbon assimilation rate per transpired water). This has been observed in response to elevated CO_2_ and drought in *Populus* spp. [29], [30]. High WUE is generally regarded as a beneficial trait in crop production [31], particularly in arid and semi-arid climates. Another functional response to altered VPD_L_ and transpiration concerns biomass allocation patterns, e.g. partitioning of dry matter into the stems compared to leaves, and consequently altered sapwood area to leaf area ratio (Huber value) ([32], Sellin *et al.* submitted ms).

The plant responses to altered RH or VPD_L_ have been studied mainly in growth-chamber experiments or using closed or open top chambers *in situ* [23], [25], [26], [33], [34]. However, free-air experiments studying the long-term effects of altered atmospheric humidity and VPD_L_ on development and growth of trees are absent. In the current paper, we present results from the free-air humidity manipulation (FAHM) experiment, where RH and water flux through deciduous tree canopy are reduced [35].

Hybrid aspen (*Populus tremula* L.×*P. tremuloides* Michx.) is used as a test tree. *Populus* spp. are widely used as model organisms among woody plants in experimental botany. Aspens have great ecological importance because a considerably large number of organisms including several endangered species is found in association with aspens and aspen forests [36], [37], [38]. Aspen species, including hybrids, have economic importance primarily as resources for pulpwood, logs and energy wood [39], [40], [41]. Several studies have previously been conducted on the responses of aspen to climate change, because their circumboreal range largely overlaps with areas where drastic climate change is predicted [1]. It has been found that increasing CO_2_ and temperature promote the growth of aspen trees, as shown by FACE experiments [42], climate models [43] and studies on growth of aspens during the 20^th^ century [44], [45]. The major negative impact of climate change on the performance of aspen trees is attributed to recurring droughts [43] and subsequent infestations by pests and diseases, witnessed by recent declines in some parts of the aspen range [46], [47].

Our aim was to analyse the growth and related functional responses of hybrid aspen trees to elevated atmospheric humidity during three growing seasons in a free air experiment. The main hypothesis was that elevated atmospheric humidity increases above-ground (stem and foliage) growth rate of the trees due to decreased transpiration.

## Materials and Methods

### Study area

**Figure 1:**
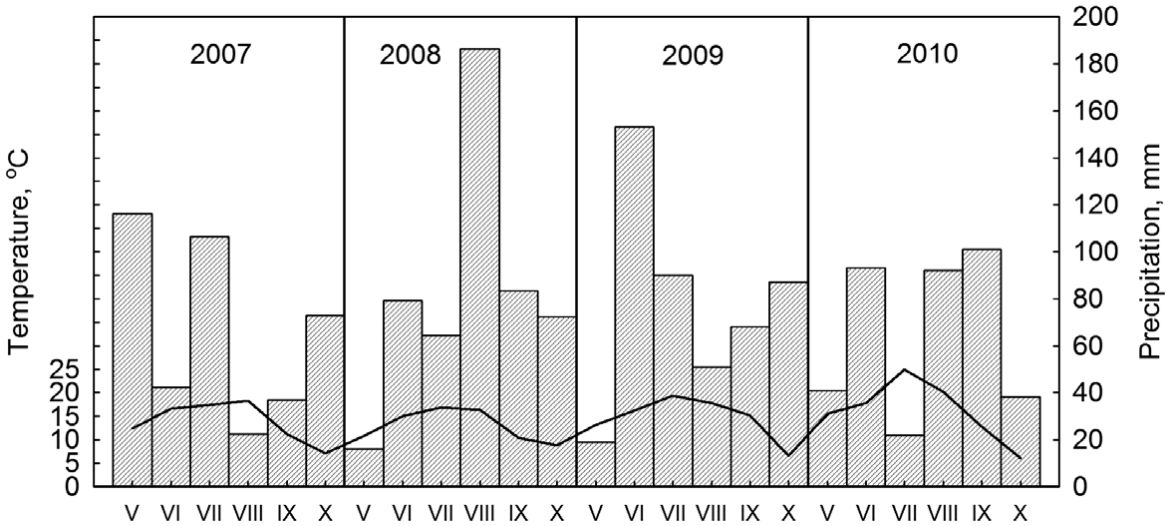
Monthly mean air temperature (line) and precipitation (columns) of the growing seasons of the study period.

The study area lies in South-East Estonia (58°14′N, 27°17′E), representing the boreo-nemoral vegetation zone and the continental temperate climate zone. The study period (2007–2010) comprises two years (2008 and 2009) with relatively high precipitation during the growing season and one year (2010) with a significant drought period in the middle of the growing season (Fig. 1, Table 1).

**Table 1:** Weather conditions expressed as daily averages in the study area during the growing seasons (May–October) of the study period (2007–2010).

Year	Growing season (days >5°C)	Photosynthetically active radiation (mmol m^−2^ s^−1^)	Net solar radiation (kW m^−2^)	Temperature (°C)	Relative humidity (%)	Precipitation (mm)
2007	197	521	0.31	17	68	397
2008	224	473	0.28	15.2	70	502
2009	210	478	0.28	17.6	65	468
2010	219	485	0.28	19.7	64	377

Soil type at the study site according to WRB is Planosol [35]. Planosols are typical in this region, where soils have developed on reddish till on Devonian sands and gleys. As for water regime, they are well-drained automorphic soils. Thus, water availability for plants depends primarily on precipitation and soil water holding capacity, which ranges from 150 to 160 mm in a 75 cm soil layer. Soil fertility estimated by total N and organic matter content can be considered good (Table 2).

**Table 2:** Chemical properties of the soil humus horizon in the experimental plots.

Plot	pH_KCl_	Total N (%)	Org. matter (%)	P (mg kg^−1^)	K (mg kg^−1^)	Ca (mg kg^−1^)	Mg (mg kg^−1^)
C1	4.1	0.12	2.67	28	57	670	96
C2	4.4	0.09	2.18	27	72	691	120
C4	4.1	0.16	3.18	32	41	571	85
H1	4.8	0.11	2.48	45	48	718	104
H2	4.3	0.12	2.67	21	52	608	114
H4	4.2	0.12	2.53	32	61	687	102
Average	4.3	0.12	2.62	31	55	657	103

### FAHM facility

**Figure 2:**
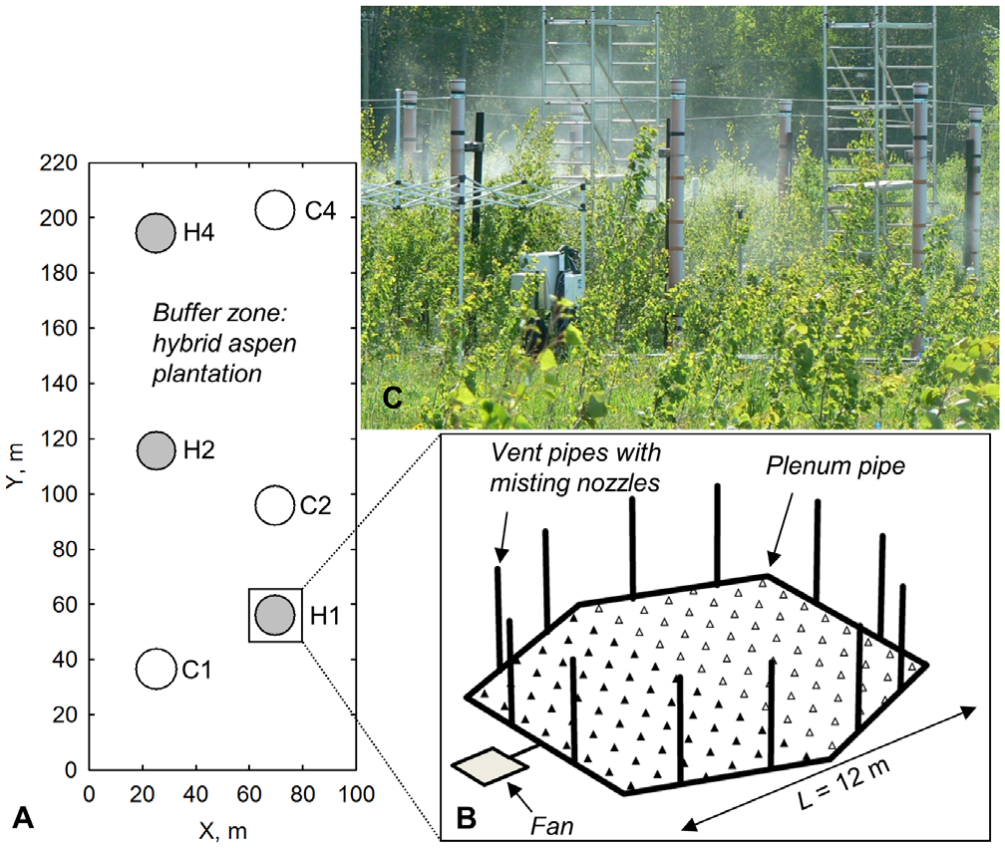
FAHM experimental area: a) locations (*X*: distance from the west edge, *Y*: distance from the south edge) of control (empty circles) and humidification plots (filled circles), b) general layout of a humidification plot comprising trees - hybrid aspens (filled triangles) and silver birches (empty triangles), *L* indicates the distance between opposite vent pipe pairs and c) photo of the humidification plot taken in July 2009.

The FAHM experimental facility (http://www.lote.ut.ee/FAHM/in-english) is a 2.7 ha fenced area, where nine circular experimental plots have been established (Fig. 2). Half of each experimental plot was planted with hybrid aspen (*Populus tremula* L.×*P. tremuloides* Michx.) and the other half with silver birch (*Betula pendula* Roth.) in the autumn of 2006. One-year-old micropropagated hybrid aspens belonging to clone C05-99-34 (according to the Finnish Plant Production Inspection Centre) were planted with 1×1 m spacing. This clone has been selected for commercial propagation from the offspring between *P. tremuloides* mother (CA2530, Canada) [48] and *P. tremula* father (clone archive No 73, Finland) (Raimo Jaatinen, personal communication, 21.05.2012).

The experimental plots are surrounded by a buffer zone, which is a hybrid aspen plantation with 2×2 m spacing. Three experimental plots act as control plots (C). In three humidity-manipulated plots (H, Fig. 2) the relative humidity of the air was raised on average 7% above the ambient level (VPD_L_ between the plant leaf interior and the atmosphere reduced on average 26% compared to the control) during a misting treatment in the growing seasons of 2008, 2009 and 2010. For this purpose a computer-controlled system was built integrating two different technologies – a misting technique to atomize/vaporise water and a FACE-like technology [49] to mix humidified air inside the plots. The remaining three plots are open-top plots with different experimental purposes and were not included in the current study. A detailed description of the FAHM facility and technology is provided by Kupper *et al.* [35].

### Description of the FAHM humidification system

The FAHM system hardware (outlines are shown in Fig. 2) consists of a high-volume blower (4 m^3^ s^−1^, 11 kW), a plenum pipe (diameter 30 cm) for air distribution, 12 extendable vertical vent pipes (VVP) for emitting air, mist emitting nozzles, electrically operated valves at each VVP for turning on/off air and water flow, and a computer system to operate valves and to communicate with the central server. A centrifugal pump (1.2 kW) takes water from a nearby pond; water is filtered through a 20 and 5 µm replaceable filter and softened with a DME demineralizer (Prominent Dosiertechnik GMBH, Heidelberg, Germany). Five button-type misting nozzles (0.5 mm; Mist Cooling Inc., Richmond, TX, USA) at each VVP atomized 4 l water per hour under a pressure of 0.8 MPa (with a mist droplet size of ∼50 µm) in 2008 and 2009 [35]. The same technical approach was used also in May 2010. Since June 2010 the humidification plots were provided with additional pumps (HPE075; Mist Cooling Inc.) and finer misting nozzles (0.15 mm; Mist Cooling Inc.), atomizing 3 l water per hour under a pressure of 7 MPa (a droplet size of ∼10 µm). Decreased size of mist droplets substantially reduced leaf wetting (dielectric leaf wetness sensors attached to Em 50 data logger; Decagon Devices, Inc., Pullman, WA) of the trees. The average time of leaf wetting was 63.6% (±7.8 SE) and 2.7% (±1.4 SE) during mist fumigation in May and June 2010, respectively. Leaf temperatures, measured with MT2 fast response temperature probes (Delta-T Devices, Burwell, UK), were lower in H plots in 2009 [35], but the difference diminished considerably after misting technology was upgraded in 2010.

### Measured variables

#### Tree growth characteristics

Tree height (*H*, cm) and stem diameter at 30 cm height (*D*, mm) of all aspen trees in experimental plots were measured after the end of each growing season. Tree height was measured with a telescopic measuring rod Nedo mEssfix-S (Nedo GmbH & Co.KG, Dornstetten, Germany), stem diameter, with a digital caliper LIMIT (Luna AB, Alingsås, Sweden). Stem volume (*V*, cm^3^) was estimated as follows:


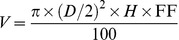
(1)

where FF is constant form factor (0.5).

Current annual increment of the growth characteristic (Δ*H*, Δ*D*, Δ*V*) was estimated as the difference in the respective characteristic measured in consecutive years. The ratio between *H* and *D* was defined as slenderness (*S*) of the tree. The ratio between Δ*V* and total leaf area was used to express the allocation pattern between stem and leaves. Arithmetic means of all estimated tree growth characteristics in each experimental plot and study year are provided in Table S1.

#### Functional traits

Functional traits characterizing tree water use and nutrient uptake by leaves were estimated during the second and third year of the experiment (2009–2010). In the first year of the experiment (2008) only growth characteristics were recorded, as trees were too small for proper mounting of analysis hardware.

#### Foliar properties

Foliar concentrations of major mineral nutrients (N, P, K, Ca and Mg) were analysed in the middle of the third and fourth growing seasons when the mass of the foliage was at its highest. The sample leaves were taken across the tree crown from 10 to 15 randomly selected sample trees from each plot and dried with a Memmert 100–800 desiccator (Memmert GmbH+Co.KG, Germany) to constant weight at 70°C. Single leaf blade area was measured with WinFOLIA ver. 5.0a (Regent Instruments Inc., Quebec, Canada) software and leaves were weighed with a KERN EW 150-3M precision balance (Kern & Sohn GmbH, Balingen, Germany) to the nearest 0.001 g. On the basis of single leaf area (*A*_Li_, cm^2^) and dry weight (*W*_Li_, g), leaf mass per area (LMA, g m^−2^) was derived.

The concentration of total N was determined by standard Kjeldahl procedure using a Kjeltec Auto 1030 Analyzer (FOSS Tecator AB, Höganäs, Sweden); P, Ca and Mg were determined spectrophotometrically from Kjeldahl digest using a FIAstar 5000 Analyzer (FOSS Tecator AB). Concentration of K was determined with Flame Photometer Model 420 (Sherwood Scientific Ltd., Cambridge, UK).

The total foliage area was determined at the beginning of August in 2009 and at the end of July in 2010. For that purpose, all leaves of the sample trees were counted and 30 to 45 sample leaves were randomly collected and their area was measured with a LI-3100C optical area meter (LI-COR Biosciences, Lincoln, NE). The total leaf area (*A*_LT_, m^2^) was calculated from the area of sampled leaves and the total number of leaves (*n*_L_). The total contents of nutrients in the foliar dry mass per ground surface area (g m^−2^) were estimated as follows (on example of nitrogen):


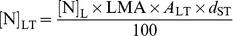
(2)

where *d*_ST_ is stand density (1 tree m^−2^).

#### Water use characteristics

The total xylem sap flow (*F*, kg) of two to four sample trees from the centre of each plot was recorded with T4.2 sap flow systems (EMS Brno, Brno, Czech Republic), fitted with ‘Baby Kucera’ sap flow sensors, from May to September in 2009 and 2010. The sap flow was measured in 20 and 22 sample trees in 2009 and 2010, respectively. The sap flow data was recorded every 1 minute and stored as 10-minute averages. The average values recorded in humid nights (RH>95%) were used in baseline subtraction procedure. Baseline values were recalculated for each 5-day interval over the whole measurement period to exclude the effect of increased stem heat storage capacity on the determination of the baseline. The sap flux density per unit projected leaf area (*F*_D_, mmol m^−2^ s^−1^) was determined as well.

Water-use efficiency (WUE, cm^3^ kg^−1^) was estimated as the ratio of stem volume increment to total sap flow (WUE = Δ*V*/*F*). Huber value (HV, m^2^ m^−2^) was defined as the ratio of stem basal area to the total leaf area (HV = *A*_base_/*A*_LT_).

#### Soil properties

In order to determine pH_KCl_, content of organic matter, and total N, P, K, Ca and Mg concentrations in the soil humus horizon, samples were taken from 10 random locations in each experimental plot in August 2009 (Table 2). Chemical analyses were conducted on air-dried samples from which visible plant and root fragments had been removed and which were sieved to <2 mm. The total N in soil samples was determined by the Kjeldahl procedure (method ISO 11261). To analyse available P, K, Ca and Mg in the soil, Mehlich 3 extractant was used. The soil pH in 1 M KCl suspensions was measured in the ratio 10 g: 25 ml using method ISO 10390. Organic matter was determined as loss on ignition (LOI, %) at 360°C.

### Data analysis

#### Notation

Our study comprises three humidification plots (treatment *x_r_* = *T* for plot *r*), and three control plots (*x_r_* = *C*), with 54–58 hybrid aspens growing in each plot (Fig. 2). The aim was to analyse the effect of the humidification treatment on multiple tree characteristics (*c*, Table 3). As a start, we performed two-tailed Student's *t*-tests to compare the trait means between C and H plots for each year, rejecting the null hypothesis of equal means when nominal *p*<0.05. Further, we needed to distinguish the effect of the treatment from that of the confounding factors. These confounders are plot-specific, but assumed to be fixed across years. We designate these *L* confounders (Table 3) for plot *r* as **w** *_r_* = {*w_r_*_,1_, …, *w_r_*_,*L*_}.

**Table 3:** Analysed variables.

Observed characteristic (*c*)	Abbreviation (unit, if recognized)	Specification level
Tree height^a^	*H* (cm)	tree
Basal diameter of stem^a^	*D* (mm)	tree
Stem volume^a^	*V* (cm^3^)	tree
Tree height increment	Δ*H* (cm yr^−1^)	tree
Stem basal diameter increment	Δ*D* (mm yr^−1^)	tree
Stem volume increment	Δ*V* (cm^3^ yr^−1^)	tree
Slenderness	*S* (m cm^−1^)	tree
Total sap flow	*F* (kg)	sample tree
Sap flux density	*F*_D_ (mmol m^−2^ s^−1^)	sample tree
Number of leaves	*n*_L_	sample tree
Total area of leaves	*A*_LT_ (m^2^)	sample tree
Huber value	HV (m^2^ m^−2^)	sample tree
Water-use efficiency	WUE (cm^3^ kg^−1^)	sample tree
Volume increment:leaf area ratio	Δ*V*: *A*_LT_ (cm^3^ m^−2^)	sample tree
Single leaf area	*A*_Li_ (cm^2^)	plot
Single leaf dry weight	*W*_Li_ (g)	plot
Leaf mass per area	LMA (g m^−2^)	plot
Foliar N concentration	[N]_L_ (%)	plot
Foliar P concentration	[P]_L_ (%)	plot
Foliar K concentration	[K]_L_ (%)	plot
Foliar Ca concentration	[Ca]_L_ (%)	plot
Foliar Mg concentration	[Mg]_L_ (%)	plot
Foliar N∶P ratio	[N:P]_L_	plot
Foliar N∶K ratio	[N:K]_L_	plot
Total N content of foliage	[N]_LT_ (g m^−2^)	plot
Total P content of foliage	[P]_LT_ (g m^−2^)	plot
Total K content of foliage	[K]_LT_ (g m^−2^)	plot
Total Ca content of foliage	[Ca]_LT_ (g m^−2^)	plot
Total Mg content of foliage	[Mg]_LT_ (g m^−2^)	plot
Explanatory variables		
Treatment (*x*)	control (*x* = *C*)humidification (*x* = *T*)	plot
*Plot-specific confounders (w)*		
Distance from the south edge of the experimental area	*Y* (m)	plot
Distance from the west edge of the experimental area	*X* (m)	plot
Soil^b^ pH_KCl_	pH_S_	plot
Soil organic matter concentration	Org_S_ (%)	plot
Soil total N concentration	[N]_S_ (%)	plot
Soil P content	[P]_S_ (mg kg^−1^)	plot
Soil K content	[K]_S_ (mg kg^−1^)	plot
Soil Ca content	[Ca]_S_ (mg kg^−1^)	plot
Soil Mg content	[Mg]_S_ (mg kg^−1^)	plot

a *H*, *D* and *V* of the previous year were used as explanatory variables while predicting the current year increments.

b soil variables characterise A-horizon.

#### Model

A linear model with nested design was employed in order to test the significance of the humidification on the observed growth traits through three study years and considering the confounding effects from the heterogeneity of the soil variables within the 2.7 ha study area.

We assume for a characteristic *c* (e.g. height) that is observed to be *y_c_*_,*i*,*r*,*t*_ for tree *i* from plot *r* on year *t* is normally distributed with the plot mean *μ_c_*_,*r*,*t*_, and trait variance *σ* 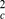: *y_c_*_,*i*,*r*,*t*_∼*N* (*μ_c_*_,*r*,*t*_, *σ* 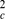). The mean of the trait in the plot depends linearly on the treatment of the plot *x_r_*, covariates **w** *_r_* and the mean of the previous year:


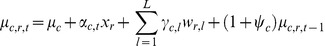
(3)

where, *α_c_*_,*t*_ is the additional effect of the treatment *x_r_* on year *t*, {*γ_c_*} are effects of plot-specific confounders, and *ψ_c_* is the contribution from the mean of the previous year with 1 added for convenience (see below). We are interested in making inferences about whether the growth differs between the two treatments. We thus model:


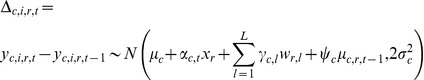
(4)

where the distribution of Δ*_c_*_,*i*,*r*,*t*_ is the difference between two normal distributions, and thus itself normal. For other traits, we do not model the yearly growth, but the trait itself; in these cases, we include the year in the model as a continuous covariate.

#### Inference and model selection

We employed stepwise model selection, adding confounders and experimental variables that improve the Akaike Information Criterion (AIC) to the model using the “step” function, using both forward and backward steps, in R software environment (http://www.r-project.org). After model selection, we tested individual explanatory variables for statistical significance by removing them from the model, calculating the log likelihoods under the two models, and assessing the significance of the change. We used the “drop1” function in R and χ^2^-test with one degree of freedom for calculating *p*-values, natural for a nested model.

To correct for multiple testing arising from assessing the effect of treatment and other variables on many plant characteristics, we calculated *q*-values, the minimal false discovery rate for which the nominal *p*-value is significant [50], for all the nested tests performed on model selection results. We considered an association to be significant if the *q*-value was less than 0.05, which means that we expect 5% false positive calls in the set of all associations we call significant.

## Results

### Above-ground growth

**Figure 3:**
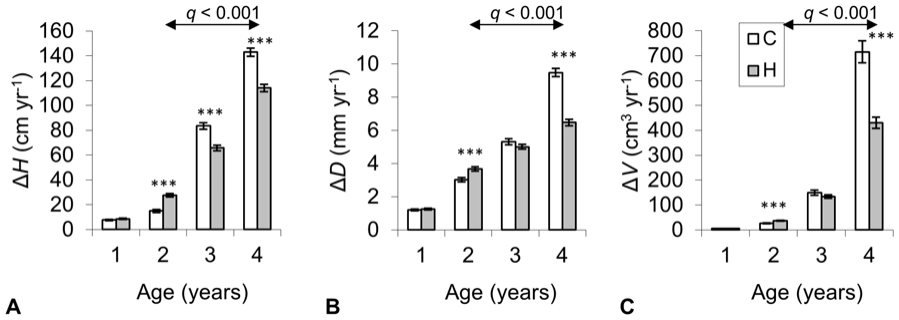
Height (a), diameter (b) and stem volume (c) increments (Δ*H*, Δ*D* and Δ*V* respectively) of hybrid aspens in control (C) and humidified (H) plots during the study period. The significance of treatment effect (*t*-test) in individual years is indicated with asterisks and *q*-values (model) show the summary effect over the years when humidification was applied (age 2–4 years). Whiskers denote ± standard error.

**Figure 4:**
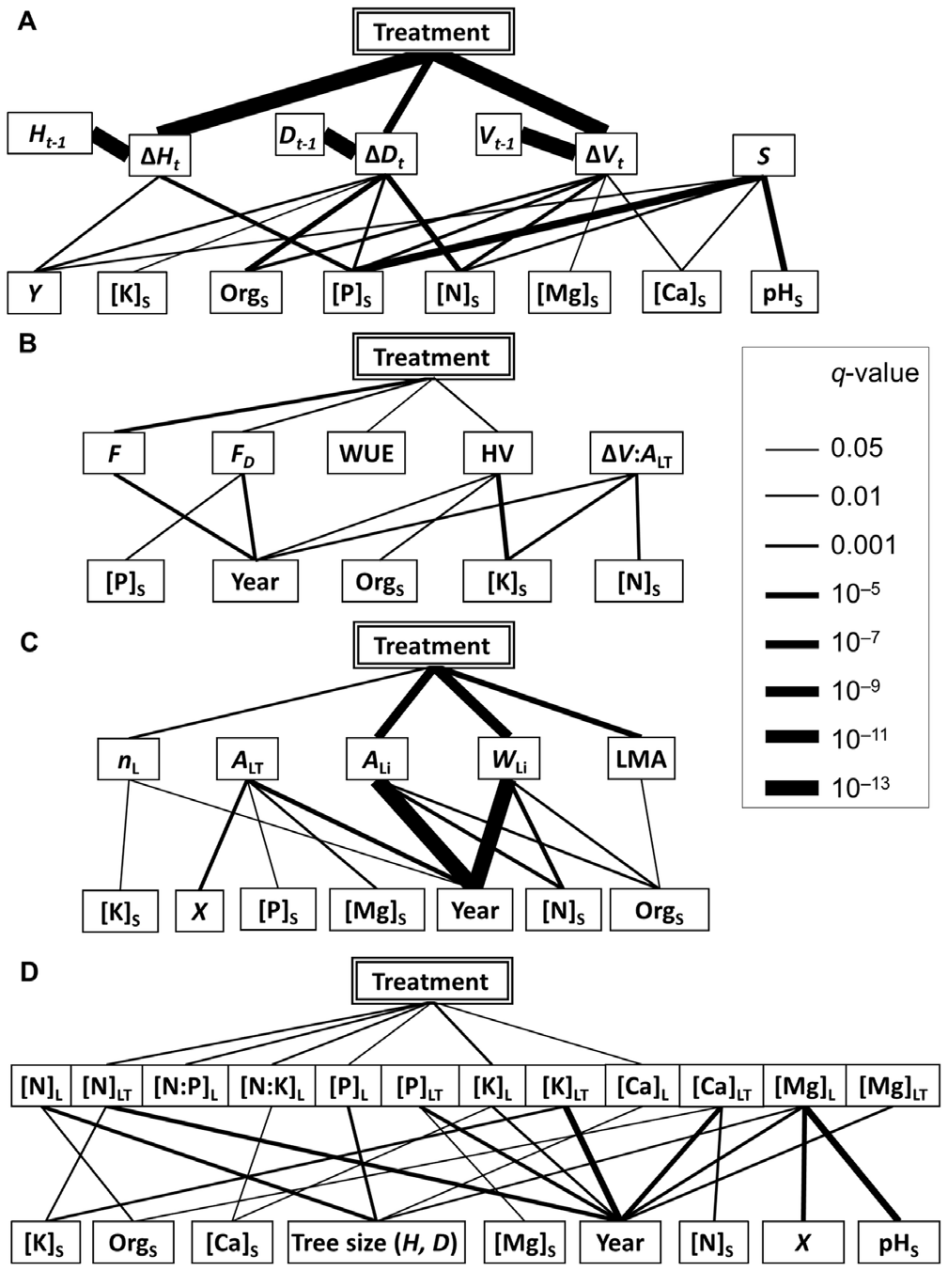
Graphical representation of the first-order relationships between treatment and confounding variables with a) tree growth, b) water use, c) leaf size and d) foliar chemistry characteristics. A solid line between two nodes denotes a significant (*q*<0.05) association between the variables, with the thickness of the line proportional to the statistical significance (−log(*q*)).

**Figure 5:**
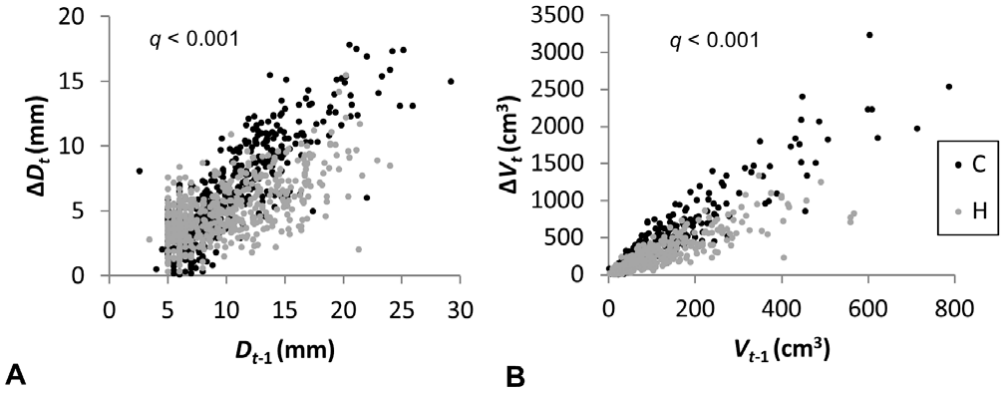
The interaction between treatment and size of the trees at the end of the previous (*t*−1) growing season for predicting the current year (*t*) increment in control (C) and humidified (H) plots: a) stem diameter (*D*), b) stem volume (*V*). Points represent individual measurements across three years of the experiment.

Pairwise comparison of the tree growth characteristics in C and H plots during the individual study years (plot means are given in Table S1) revealed that the growth rate in H plots was higher during the first year of the experiment but remained significantly lower compared to C plots after the second and third year (Fig. 3). The treatment effect on growth was highly significant (*q*<0.01) also across the whole study period (Fig. 4a). Additionally, tree growth increment in the given year was always strongly dependent on the size of the tree (i.e. the value of the given trait at the end of the previous growing period). The studied tree characteristics were significantly affected also by soil variables, with the strongest effects from [N]_S_, [P]_S_ and Org_S_. There existed a significant interaction between treatment and the size of the tree at the end of previous growing season for predicting the current year increment. Stems of the trees of the same size grew slower in H plots compared to C plots, while such a distinction was greater in bigger trees (Fig. 5).

### Water use and allocation patterns

**Figure 6:**
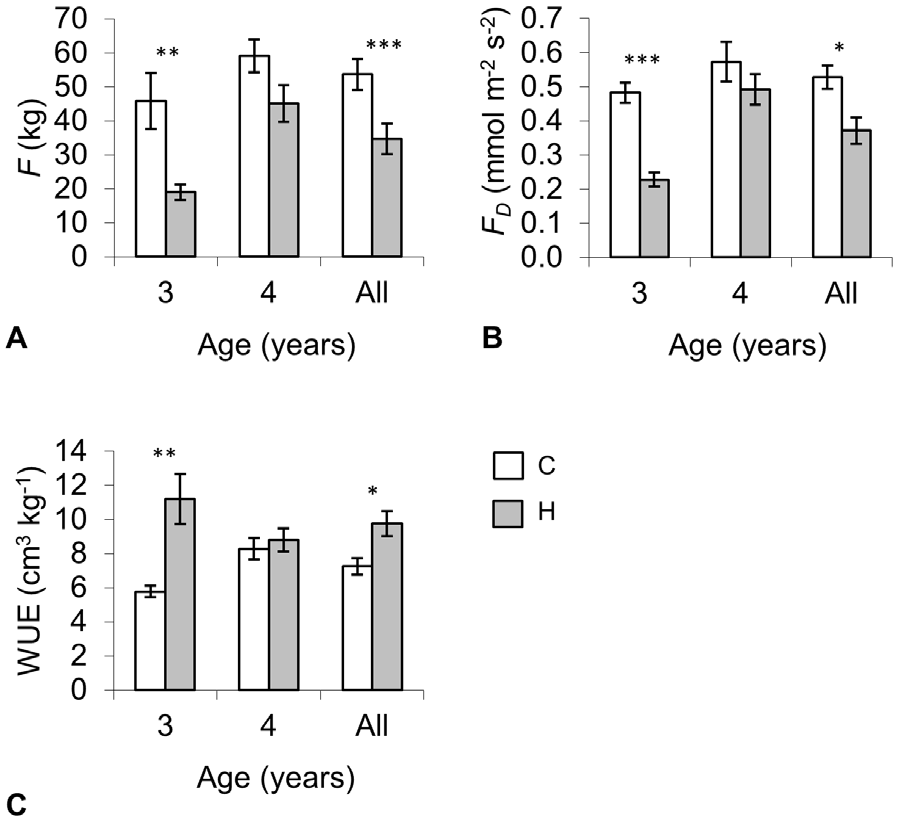
Comparison of physiological traits: a) *F* – total sap flow, b) *F_D_* – sap flux density, c) WUE – water-use efficiency of hybrid aspens at the ages of 3 and 4 years in control (C) and humidified (H) plots (*t*-test) and summary effect of the treatment over two years (model), whiskers denote ± standard error.

The distinction in water use characteristics was more pronounced during the third growing season, with almost two times lower estimates of sap flow (*F*) and sap flux density (*F*_D_) in H plots (Fig. 6). At the same time water-use efficiency (WUE) was higher in H plots. Across the whole study period, *F* was more strongly affected by the treatment than *F*_D_ (Fig. 4b). Both *F* and *F*_D_ varied between the study years, whereas *F*_D_ was strongly affected by the year×treatment interaction (Table S2).

The mean slenderness (*S*) of the aspen stems was 1.4±0.01 m cm^−1^ and it did not vary between the treatments nor the study years. Huber value depended on the year and treatment (Fig. 4), although the differences in mean values were small: 3.27±0.14×10^−2^ m^2^ m^−2^ in H plots and 3.46±0.21×10^−2^ m^2^ m^−2^ in C plots. The stem volume increment to leaf area ratio was invariant between C and H plots but depended on the year being 290±14 cm^3^ m^−2^ in 2009 and 360±24 cm^3^ m^−2^ in 2010.

### Foliar characteristics

Humidification treatment had a significant effect on various foliar characteristics (Table 4, Fig. 4c, 4d). The strongest effects during the study years included the smaller size (*A*_Li_ and *W*_Li_) and LMA of individual leaves, whereas *A*_LT_ was invariant because of higher *n*_L_ in H plots (Fig. 4c). Total N content of the foliage ([N]_LT_) and foliar N∶P and N∶K ratios were lower in H plots (Table 4, Fig. 4d). The variation in the contents and concentrations of foliar nutrients depended strongly on study year and tree size (Fig. 4d). The treatment effect on foliar nutrient concentrations differed between the study years (Table 4) and most of these effects were marginally significant (*q*<0.05) through both study years except [K]_L_, which was substantially (*q*<0.01) higher in H plots (Fig. 4d). The confounding effect of soil chemical properties was generally weaker on foliar nutrients than on leaf size characteristics and above-ground growth rate of trees.

**Table 4:** Comparison of foliar characteristics (mean ± standard error) in control (C) and humidified (H) plots during individual study years (asterisks indicate *p*-values based on *t*-test: 0.01<*p*<0.05*; 0.001<*p*<0.01**; *p*<0.001***) and summary effect of the treatment through the years: “True” if *q*<0.05.

Foliar characteristic	Age (year)				Treatment effect
	3 (2009)		4 (2010)		
	C	H	C	H	
Number of leaves	455±38	575±36*	597±51	693±47	True
Total leaf area (m^2^)	0.88±0.12	0.73±0.08	1.44±0.09	1.16±0.09*	False
Single leaf area (cm^2^)	13.3±0.3	11.3±0.3***	26.5±0.9	19.2±0.8***	True
Single leaf weight (g)	0.12±0.003	0.09±0.003***	0.23±0.008	0.16±0.008***	True
LMA (g m^−2^)	87.9±0.9	83.5±0.9***	87.4±0.9	82.8±1.0***	True
[N]_L_ (%)	2.04±0.04	2.21±0.05*	2.44±0.03	2.17±0.05***	False
[P]_L_ (%)	0.18±0.01	0.20±0.01	0.21±0.01	0.21±0.01	True
[K]_L_ (%)	0.58±0.01	0.67±0.02**	0.68±0.02	0.75±0.05	True
[Ca]_L_ (%)	1.42±0.12	1.90±0.09**	1.99±0.17	1.97±0.11	True
[Mg]_L_ (%)	0.28±0.01	0.41±0.02***	0.28±0.01	0.33±0.02*	False
[N:P]_L_	11.22±0.40	11.10±0.27	11.94±0.35	10.38±0.37*	True
[N:K]_L_	3.56±0.13	3.32±0.09	3.62±0.13	2.96±0.23*	True
[N]_LT_ (g m^−2^)	1.55±0.32	1.41±1.18	3.08±0.29	2.09±0.23*	True
[P]_LT_ (g m^−2^)	0.14±0.03	0.13±0.02	0.26±0.03	0.20±0.02	False
[K]_LT_ (g m^−2^)	0.43±0.08	0.43±0.06	0.84±0.06	0.70±0.06	False
[Ca]_LT_ (g m^−2^)	1.06±0.20	1.19±0.13	2.58±0.44	1.87±0.18	False
[Mg]_LT_ (g m^−2^)	0.20±0.03	0.26±0.02	0.36±0.04	0.33±0.05	False

## Discussion

In the present study we reported the results concerning the growth and related functional traits of hybrid aspens in elevated atmospheric humidity conditions during the first three years of the FAHM experiment. The results are unique, as the growth of neither aspens nor other woody species has been studied under such conditions in a free air experiment.

Generally hybrid aspens at the FAHM site had grown up to 50% faster than recorded in conventional plantations established on similar abandoned agricultural soils (Planosols) in the region [51]. Faster growth rate observed at the FAHM site is probably due to reduced weed competition, as weed control was carried out during the first two years at the FAHM site and obviously also due to closer spacing in the FAHM plots (1×1 m) compared to the average spacing in commercial plantations (3×4 m). Closer spacing has been found to significantly promote height growth of young hybrid aspens [52] and other deciduous trees [53].

Our main hypothesis regarding the expected improvement in growth rate of the above-ground part (stem and leaves) of hybrid aspens in response to elevated humidity was not supported by the results when considering the whole study period (Figs. 3 and 4a). Although the trees growing in H plots were more successful in overcoming the post-planting stress in 2008, the growth rate remained considerably slower during the following two study years. Similar trends were observed in silver birches in the FAHM experiment (Sellin *et al.* submitted ms). The modeling results indicated that the size of the aspen trees at the end of the previous growing season was always a good predictor of growth increment in the current year (Fig. 4a). This is in accordance with other studies modeling growth of individual trees [54], [55], [56]. At the same time, we found a strong interaction between the treatment effect and tree size on current year increment (Fig. 5). As a rule, bigger trees are in a better position to exploit resources, which means that they can grow faster, but trees growing under elevated humidity conditions were less successful in transferring their size benefit to growth increment. This could indicate that trees were more strongly affected by competition in H plots, although generally we would expect only minor impact from competition at the given stand density and age. High atmospheric humidity caused also some developmental retardation of foliage, resulting in smaller leaf blade area and dry weight (Table 4). Thus we contradicted with previous studies where herbaceous plants or young tree saplings had larger leaves under low VPD_L_ [25], [26]. As a compensatory response to reduced single leaf area, the number of leaves increased, thus the reduction in total leaf area in H plots was not significant across the whole study period. The humidification caused a small but significant increase in sapwood-to-leaf area ratio of stems (HV). However, this change should rather improve tree water potential and growth and does not explain the decreased growth in our study.

Despite the general homogeneity of the soil conditions at the experimental site, there existed certain variability in topsoil nutrients and pH (Table 2), which had some impact on tree growth rates in particular plots (Fig. 4a). All studied growth traits were affected by [P]_S_, which has been shown as an important growth factor in hybrid aspen plantations on abandoned agricultural lands in Estonia [51]. However, the impact of humidification on growth remained highly significant also after considering the side effects from soil-related confounders.

As expected, the average xylem sap flow was reduced by 35% and sap flux density by 30% in H plots (Fig. 6), in agreement with previous observations in natural conditions under lower VPD_L_ [16], [23], [57], [58]. Despite of decreased growth, WUE increased in H plots (Fig. 6). Generally increase in WUE is observed when transpiration is low, e.g. in response to drought and elevated CO_2_ [29], [30]. Therefore the reduction in growth rate of the trees cannot be directly attributed to changes in WUE.

A possible explanation for lower growth under elevated humidity is hindered acquisition of mineral nutrients. Nutrients supplied to plant roots by mass flow include NO_3_^−^, Ca^+2^ and Mg^+2^, thus, considering the transpiration-driven mass flow concept [59], we could expect lowered concentrations or total contents of these nutrients in the leaves of the humidity-treated trees. In H plots [N]_LT_ was lower compared to C plots in both study years and only [N]_LT_ and none of the other studied total foliar nutrient contents was significantly affected by the humidification manipulation (Fig. 4d). At the same time [N]_L_ showed reversed trends in different study years (Table 4). The critical limit of [N]_L_ for fast growth was 2.4% in 7-yr-old hybrid aspen plantations [60]. In the current study [N]_L_ was below this limit in both C and H plots in 2009 but remained below the limit only in H plots in 2010. Foliar N∶P and N∶K ratios were lower in H plots, however these shifts were marginally significant. Although the estimated foliar N∶P ratios in C and H plots fell into the sufficient range (10–20) of these elements [61], they were close to lower border indicating slight N deficiency in H plots. Obviously for hybrid aspen, representing a fast-growing hardwood species, even slight deviation from optimal N-uptake conditions could cause considerable growth retardation. [Ca]_LT_, [Mg]_LT_ and [Mg]_L_ were not significantly affected by the treatment, whereas [Ca]_L_ tended to be higher in H plots. Thus these elements were not limiting aspen growth. Moreover, Cramer *et al.* [59] suggest that transpiration-driven mass flow is particularly associated with N uptake. Foliar potassium concentration turned out to be higher in H plots. K^+^ uptake is less affected by mass flow as it moves to roots primarily via diffusion. Most of the studied foliar chemistry characteristics were associated with tree size and year, indicating the importance of tree age and climate of the study year on these traits.

Leaf dry mass per area (LMA) was significantly lower in H plots and this was one of the strongest effects from the treatment. Lower LMA generally refers to lower foliar photosynthetic capacity [62], [63]. Thus lower values of LMA and N uptake indicate that lower photosynthetic capacity was one of the reasons causing lower growth rate of the trees in humidified plots.

While interpreting the results from the first two years of the FAHM experiment we must also consider potential side effect of leaf wetting which resulted in 2.4°C lower leaf temperatures in silver birches in H plots during the misting in 2009 [35]. However, the leaf wetting was reduced and the difference in leaf temperatures diminished after upgrading the misting technology since 2010 (see “Description of the FAHM humidification system”), whereas the distinction in growth increments between C and H plots became even bigger in 2010 (Fig. 3). As 2010 was the warmest year within the study period (Table 1), the growth reduction in H could not be caused by lower leaf temperature. Hence, decreased transpiration and sap flow, not decreased temperature due to leaf wetting, seems to be responsible for decreased growth of hybrid aspen in humid free air conditions.

To summarise, the obtained results refer to the decreased production potential of hybrid aspen trees in H plots. We attribute this primarily to reduced N uptake due to decreased transpiration-driven mass flow of NO_3_^−^ in soil. The expected climate-change-induced increase in the growth rate of trees at northern latitudes (boreal areas) is associated rather with an earlier start of the growing season in spring than with increasing atmospheric CO_2_ levels, i.e. the primary drivers are increasing soil and air temperatures [64]. Our results suggest that the potential growth improvement could be smaller than expected if temperature rise is accompanied by a rise in atmospheric humidity.

## Supporting Information

Table S1 **Growth characteristics (arithmetic mean ± standard error) of hybrid aspens in control (C1, C2, C4) and humidified (H1, H2, H4) plots during the study period.**
(DOC)

Table S2 **First and second order results of the model.**
(DOC)
