# Supplementary material for: Climate Change at Northern Latitudes: Rising Atmospheric Humidity Decreases Transpiration, N-Uptake and Growth Rate of Hybrid Aspen
Source: PLoS One. 2012 Aug 6;7(8):e42648. doi: 10.1371/journal.pone.0042648 (PMC3412825; doi:10.1371/journal.pone.0042648)
Supplement: Table S1 — Growth characteristics (arithmetic mean ± standard error) of hybrid aspens in control (C1, C2, C4) and humidified (H1, H2, H4) plots during the study period. (DOC) [file pone.0042648.s001.doc]

Table S1. Growth characteristics (arithmetic mean ± standard error) of hybrid aspens in control (C1, C2, C4) and humidified (H1, H2, H4) plots during the study period

| Year | Plot | *n* | *H*  (cm) | *D*  (mm) | *V*  (cm^3^) | Δ*H*  (cm yr^–1^) | Δ*D*  (mm yr^–1^) | Δ*V*  (cm^3^ yr^–1^) |
| --- | --- | --- | --- | --- | --- | --- | --- | --- |
| 2007 | C1 | 58 | 110±1.3 | 6.2±0.10 | 17±0.7 | 5±1.3 | 1.0±0.10 | 6±0.6 |
| 2007 | C2 | 58 | 102±1.2 | 6.0±0.10 | 15±0.6 | 7±1.3 | 1.0±0.11 | 5±0.6 |
| 2007 | C4 | 57 | 105±1.5 | 6.2±0.11 | 16±0.8 | 3±1.1 | 1.0±0.08 | 5±0.5 |
| 2007 | H1 | 54 | 106±1.5 | 6.0±0.12 | 15±0.7 | 7±1.7 | 0.9±0.09 | 5±0.5 |
| 2007 | H2 | 56 | 105±1.6 | 6.0±0.10 | 15±0.7 | 6±1.1 | 1.1±0.09 | 6±0.5 |
| 2007 | H4 | 57 | 105±1.4 | 6.1±0.11 | 16±0.7 | 6±1.1 | 1.1±0.10 | 6±0.7 |
| 2008 | C1 | 58 | 118±1.6 | 8.8±0.14 | 37±1.5 | 7±1.5 | 2.6±0.15 | 20±1.4 |
| 2008 | C2 | 57 | 108±2.3 | 8.2±0.23 | 31±2.2 | 6±2.2 | 2.3±0.21 | 17±2.1 |
| 2008 | C4 | 57 | 128±2.4 | 10.2±0.28 | 57±4.1 | 23±2.4 | 4.0±0.25 | 41±3.8 |
| 2008 | H1 | 54 | 129±2.9 | 9.3±0.26 | 48±3.3 | 23±2.9 | 3.4±0.25 | 33±3.1 |
| 2008 | H2 | 56 | 132±2.9 | 9.8±0.27 | 54±4.2 | 27±2.6 | 3.7±0.22 | 39±3.8 |
| 2008 | H4 | 57 | 133±2.8 | 9.9±0.24 | 55±3.6 | 28±2.5 | 3.8±0.21 | 39±3.4 |
| 2009 | C1 | 58 | 201±4.1 | 13.5±0.32 | 154±10.2 | 83±3.6 | 4.7±0.23 | 117±9.2 |
| 2009 | C2 | 57 | 170±5.4 | 12.2±0.33 | 112±8.7 | 62±4.9 | 4.0±0.24 | 81±7.5 |
| 2009 | C4 | 57 | 229±5.4 | 17.4±0.59 | 306±25.0 | 101±4.5 | 7.2±0.35 | 249±21.7 |
| 2009 | H1 | 54 | 187±5.1 | 13.8±0.38 | 154±11.9 | 58±3.3 | 4.4±0.20 | 106±9.4 |
| 2009 | H2 | 56 | 192±5.6 | 14.8±0.44 | 186±16.5 | 61±3.8 | 5.0±0.27 | 132±13.1 |
| 2009 | H4 | 57 | 212±5.3 | 15.4±0.41 | 217±15.2 | 78±4.1 | 5.5±0.22 | 162±12.2 |
| 2010 | C1 | 57 | 338±5.4 | 22.8±0.62 | 747±49.5 | 138±3.6 | 9.4±0.35 | 595±40.7 |
| 2010 | C2 | 57 | 290±7.1 | 20.0±0.59 | 504±36.6 | 120±4.3 | 7.8±0.32 | 392±29.0 |
| 2010 | C4 | 57 | 400±8.7 | 28.6±0.98 | 1466±119.8 | 171±6.3 | 11.3±0.47 | 1160±96.7 |
| 2010 | H1 | 54 | 316±7.5 | 20.7±0.66 | 598±51.0 | 129±4.2 | 7.0±0.36 | 444±40.7 |
| 2010 | H2 | 55 | 282±8.0 | 20.6±0.68 | 540±49.5 | 90±4.5 | 5.8±0.31 | 353±34.8 |
| 2010 | H4 | 57 | 335±7.9 | 22.0±0.67 | 712±53.2 | 123±4.7 | 6.6±0.31 | 495±39.6 |
