# Supplementary material for: Climate Change at Northern Latitudes: Rising Atmospheric Humidity Decreases Transpiration, N-Uptake and Growth Rate of Hybrid Aspen
Source: PLoS One. 2012 Aug 6;7(8):e42648. doi: 10.1371/journal.pone.0042648 (PMC3412825; doi:10.1371/journal.pone.0042648)
Supplement: Table S2 — First and second order results of the model. (DOC) [file pone.0042648.s002.doc]

Table S2. First and second order results of the model

| Response and explanatory variables | Df | Sum of Sq | RSS | AIC | Pr(Chi) | *q* |
| --- | --- | --- | --- | --- | --- | --- |
| Height increment of the current year (Δ*H_t_*), first order |  |  |  |  |  |  |
| <none> |  |  | 863159 | 4814.1 |  |  |
| *H_t_*_–1_ | 1 | 436251 | 1299410 | 5086.6 | <0.0001 | <0.0001 |
| Treatment | 1 | 134148 | 997308 | 4909 | <0.0001 | <0.0001 |
| [P]_S_ | 1 | 22411 | 885571 | 4829.3 | <0.0001 | 0.0002 |
| *Y* | 1 | 12791 | 875951 | 4822 | 0.0017 | 0.0043 |
| Height increment of the current year (Δ*H_t_*), second order | Df | Sum of Sq | RSS | AIC | Pr(Chi) | *q* |
| <none> |  |  | 814281 | 4783 |  |  |
| *H_t_*_–1_ | 1 | 2156.4 | 816437 | 4782.7 | 0.1828 | 0.1908 |
| Treatment | 1 | 4520.2 | 818801 | 4784.7 | 0.0539 | 0.0732 |
| [P]_S_ | 1 | 11990.4 | 826271 | 4790.8 | 0.0017 | 0.0043 |
| *Y* | 1 | 8073.4 | 822354 | 4787.6 | 0.0101 | 0.0181 |
| pH_S_ | 1 | 23863.6 | 838144 | 4800.3 | <0.0001 | 0.0001 |
| *X* | 1 | 16734.7 | 831015 | 4794.6 | 0.0002 | 0.0008 |
| *H_t_*_–1_*[P]_S_ | 1 | 18208.9 | 832490 | 4795.8 | 0.0001 | 0.0005 |
| Treatment*[P]_S_ | 1 | 17061.6 | 831342 | 4794.9 | 0.0002 | 0.0007 |
| Diameter increment of the current year (Δ*D_t_*), first order | Df | Sum of Sq | RSS | AIC | Pr(Chi) | *q* |
| <none> |  |  | 2600.7 | 929.04 |  |  |
| *D_t_*_-1_ | 1 | 2573.62 | 5174.3 | 1388.64 | <0.0001 | <0.0001 |
| Treatment | 1 | 129.25 | 2729.9 | 959.59 | <0.0001 | <0.0001 |
| [Ca]_S_ | 1 | 15.83 | 2616.5 | 931.11 | 0.0436 | 0.0617 |
| [P]_S_ | 1 | 44.99 | 2645.6 | 938.55 | 0.0007 | 0.0021 |
| [N]_S_ | 1 | 96.61 | 2697.3 | 951.51 | <0.0001 | <0.0001 |
| Org_S_ | 1 | 88.27 | 2688.9 | 949.44 | <0.0001 | <0.0001 |
| *Y* | 1 | 39.73 | 2640.4 | 937.21 | 0.0014 | 0.0038 |
| [K]_S_ | 1 | 23.21 | 2623.9 | 933 | 0.0146 | 0.0250 |
| pH_S_ | 1 | 9.75 | 2610.4 | 929.55 | 0.1130 | 0.1311 |
| Diameter increment of the current year (Δ*D_t_*), second order | Df | Sum of Sq | RSS | AIC | Pr(Chi) | *q* |
| <none> |  |  | 2413.7 | 870.99 |  |  |
| *D_t_*_-1_ | 1 | 2674 | 5087.7 | 1369.32 | <0.0001 | <0.0001 |
| Treatment | 1 | 3.96 | 2417.7 | 870.09 | 0.2945 | 0.3047 |
| [Ca]_S_ | 1 | 73.15 | 2486.9 | 889.02 | <0.0001 | 0.0001 |
| *D_t_*_-1_*Treatment | 1 | 277.96 | 2691.7 | 942.12 | <0.0001 | <0.0001 |
| Treatment*[Ca]_S_ | 1 | 39.89 | 2453.6 | 879.99 | 0.0009 | 0.0026 |
| Volume increment of the current year (Δ*V_t_*), first order | Df | Sum of Sq | RSS | AIC | Pr(Chi) | *q* |
| <none> |  |  | 18858671 | 6893.5 |  |  |
| *V_t_*_-1_ | 1 | 75512594 | 94371265 | 7972 | <0.0001 | <0.0001 |
| Treatment | 1 | 1989670 | 20848340 | 6958.8 | <0.0001 | <0.0001 |
| [N]_S_ | 1 | 426458 | 19285128 | 6906.5 | 0.0001 | 0.0005 |
| *X* | 1 | 64285 | 18922956 | 6893.8 | 0.1308 | 0.1434 |
| [Ca]_S_ | 1 | 199033 | 19057704 | 6898.6 | 0.0080 | 0.0150 |
| [P]_S_ | 1 | 358639 | 19217310 | 6904.2 | 0.0004 | 0.0012 |

Table S2 (continued).

| Org_S_ | 1 | 327614 | 19186285 | 6903.1 | 0.0007 | 0.0021 |
| --- | --- | --- | --- | --- | --- | --- |
| [Mg]_S_ | 1 | 156747 | 19015418 | 6897.1 | 0.0184 | 0.0300 |
| *Y* | 1 | 91061 | 18949731 | 6894.8 | 0.0722 | 0.0928 |
| Volume increment of the current year (Δ*V_t_*), second order | Df | Sum of Sq | RSS | AIC | Pr(Chi) | *q* |
| <none> |  |  | 13905689 | 6689.1 |  |  |
| *V_t_*_-1_ | 1 | 718768 | 14624457 | 6720.9 | <0.0001 | <0.0001 |
| Treatment | 1 | 10774 | 13916463 | 6687.6 | 0.4710 | 0.4790 |
| [P]_S_ | 1 | 293790 | 14199479 | 6701.1 | 0.0002 | 0.0007 |
| [Ca]_S_ | 1 | 122121 | 14027811 | 6693 | 0.0154 | 0.0261 |
| pH_S_ | 1 | 311157 | 14216846 | 6701.9 | 0.0001 | 0.0005 |
| *V_t_*_-1_*Treatment | 1 | 4085889 | 17991578 | 6859.9 | <0.0001 | <0.0001 |
| *V_t_*_-1_*[P]_S_ | 1 | 337363 | 14243053 | 6703.2 | 0.0001 | 0.0003 |
| *V_t_*_-1_*[Mg]_S_ | 1 | 103136 | 14008826 | 6692.1 | 0.0260 | 0.0393 |
| [P]_S_*pH_S_ | 1 | 299646 | 14205335 | 6701.4 | 0.0002 | 0.0007 |
| Slenderness (*S*), first order | Df | Sum of Sq | RSS | AIC | Pr(Chi) | *q* |
| <none> |  |  | 22.015 | -2280.8 |  |  |
| [P]_S_ | 1 | 1.1669 | 23.181 | -2248.2 | <0.0001 | <0.0001 |
| *Y* | 1 | 0.24113 | 22.256 | -2275.5 | 0.0069 | 0.0134 |
| pH_S_ | 1 | 0.89704 | 22.912 | -2256.1 | <0.0001 | <0.0001 |
| [N]_S_ | 1 | 0.28787 | 22.302 | -2274.1 | 0.0032 | 0.0074 |
| [Ca]_S_ | 1 | 0.22532 | 22.24 | -2276 | 0.0090 | 0.0166 |
| Slenderness (*S*), second order | Df | Sum of Sq | RSS | AIC | Pr(Chi) | *q* |
| <none> |  |  | 21.947 | -2280.9 |  |  |
| [P]_S_ | 1 | 1.20569 | 23.152 | -2247 | <0.0001 | <0.0001 |
| *Y* | 1 | 0.27163 | 22.218 | -2274.7 | 0.0041 | 0.0089 |
| pH_S_ | 1 | 0.14171 | 22.088 | -2278.6 | 0.0377 | 0.0549 |
| [N]_S_ | 1 | 0.33553 | 22.282 | -2272.7 | 0.0014 | 0.0038 |
| [Ca]_S_ | 1 | 0.06013 | 22.007 | -2281.1 | 0.1754 | 0.1839 |
| pH_S_*[Ca]_S_ | 1 | 0.06795 | 22.015 | -2280.8 | 0.1498 | 0.1577 |
| Total sap flow (*F*), first order | Df | Sum of Sq | RSS | AIC | Pr(Chi) | *q* |
| <none> |  |  | 7639 | 198.5 |  |  |
| Year | 1 | 4052.2 | 11691.2 | 211.39 | 0.0001 | 0.0005 |
| Treatment | 1 | 3559.1 | 11198.1 | 209.88 | 0.0003 | 0.0009 |
| *Y* | 1 | 778.5 | 8417.4 | 199.9 | 0.0653 | 0.0863 |
| [Ca]_S_ | 1 | 696.5 | 8335.5 | 199.55 | 0.0805 | 0.0997 |
| Total sap flow (*F*), second order | Df | Sum of Sq | RSS | AIC | Pr(Chi) | *q* |
| <none> |  |  | 7639 | 198.5 |  |  |
| Year | 1 | 4052.2 | 11691.2 | 211.39 | 0.0001 | 0.0005 |
| Treatment | 1 | 3559.1 | 11198.1 | 209.88 | 0.0003 | 0.0009 |
| *Y* | 1 | 778.5 | 8417.4 | 199.9 | 0.0653 | 0.0863 |
| [Ca]_S_ | 1 | 696.5 | 8335.5 | 199.55 | 0.0805 | 0.0997 |
| Sap flux density (*F*_D_), first order | Df | Sum of Sq | RSS | AIC | Pr(Chi) | *q* |
| <none> |  |  | 0.54245 | -137.84 |  |  |
| Year | 1 | 0.26571 | 0.80816 | -125.89 | 0.0002 | 0.0007 |
| Treatment | 1 | 0.1147 | 0.65715 | -133.13 | 0.0096 | 0.0175 |
| [P]_S_ | 1 | 0.12897 | 0.67142 | -132.38 | 0.0063 | 0.0128 |

Table S2 (continued).

| Sap flux density (*F*_D_), second order | Df | Sum of Sq | RSS | AIC | Pr(Chi) | *q* |
| --- | --- | --- | --- | --- | --- | --- |
| <none> |  |  | 0.35293 | -146.89 |  |  |
| Year | 1 | 0.093957 | 0.44688 | -140.63 | 0.0041 | 0.0090 |
| Treatment | 1 | 0.10955 | 0.46248 | -139.43 | 0.0021 | 0.0051 |
| [P]_S_ | 1 | 0.085471 | 0.4384 | -141.3 | 0.0059 | 0.0122 |
| Year*Treatment | 1 | 0.109431 | 0.46236 | -139.44 | 0.0021 | 0.0051 |
| Year*[P]_S_ | 1 | 0.085556 | 0.43848 | -141.29 | 0.0058 | 0.0123 |
| Treatment*[P]_S_ | 1 | 0.022142 | 0.37507 | -146.76 | 0.1445 | 0.1535 |
| Number of leaves (*n*_L_), first order | Df | Sum of Sq | RSS | AIC | Pr(Chi) | *q* |
| <none> |  |  | 511895 | 345.67 |  |  |
| Treatment | 1 | 139781 | 651677 | 352.12 | 0.0037 | 0.0082 |
| [K]_S_ | 1 | 78938 | 590833 | 348.69 | 0.0251 | 0.0386 |
| Year | 1 | 78104 | 590000 | 348.64 | 0.0258 | 0.0393 |
| *X* | 1 | 36266 | 548162 | 346.06 | 0.1217 | 0.1365 |
| Number of leaves (*n*_L_), second order | Df | Sum of Sq | RSS | AIC | Pr(Chi) | *q* |
| <none> |  |  | 511895 | 345.67 |  |  |
| Treatment | 1 | 139781 | 651677 | 352.12 | 0.0037 | 0.0082 |
| [K]_S_ | 1 | 78938 | 590833 | 348.69 | 0.0251 | 0.0386 |
| Year | 1 | 78104 | 590000 | 348.64 | 0.0258 | 0.0393 |
| *X* | 1 | 36266 | 548162 | 346.06 | 0.1217 | 0.1365 |
| Total area of leaves (*A*_LT_), first order | Df | Sum of Sq | RSS | AIC | Pr(Chi) | *q* |
| <none> |  |  | 211608075 | 556.52 |  |  |
| Year | 1 | 168700066 | 380308141 | 575.04 | <0.0001 | <0.0001 |
| *X* | 1 | 97797650 | 309405725 | 567.82 | 0.0003 | 0.0009 |
| [Mg]_S_ | 1 | 80520403 | 292128478 | 565.81 | 0.0008 | 0.0023 |
| [P]_S_ | 1 | 34881516 | 246489591 | 559.86 | 0.0208 | 0.0332 |
| Total area of leaves (*A*_LT_), second order | Df | Sum of Sq | RSS | AIC | Pr(Chi) | *q* |
| <none> |  |  | 193234189 | 555.34 |  |  |
| Year | 1 | 2481 | 193236670 | 553.34 | 0.9831 | 0.9831 |
| *X* | 1 | 102226279 | 295460468 | 568.2 | 0.0001 | 0.0005 |
| [Mg]_S_ | 1 | 78344915 | 271579104 | 565.25 | 0.0006 | 0.0018 |
| [P]_S_ | 1 | 18391994 | 211626182 | 556.52 | 0.0744 | 0.0941 |
| Year*[P]_S_ | 1 | 18373886 | 211608075 | 556.52 | 0.0746 | 0.0938 |
| HUBER value (HV), first order | Df | Sum of Sq | RSS | AIC | Pr(Chi) | *q* |
| <none> |  |  | 20.073 | -31.731 |  |  |
| [K]_S_ | 1 | 9.76 | 29.833 | -14.315 | <0.0001 | 0.0001 |
| Year | 1 | 3.3353 | 23.408 | -26.199 | 0.0061 | 0.0125 |
| Org_S_ | 1 | 3.0092 | 23.082 | -26.886 | 0.0089 | 0.0166 |
| Treatment | 1 | 3.2734 | 23.346 | -26.328 | 0.0065 | 0.0131 |
| [Ca]_S_ | 1 | 1.4019 | 21.474 | -30.423 | 0.0689 | 0.0895 |
| HUBER value (HV), second order | Df | Sum of Sq | RSS | AIC | Pr(Chi) | *q* |
| <none> |  |  | 17.637 | -34.07 |  |  |
| [K]_S_ | 1 | 4.4848 | 22.122 | -24.968 | 0.0009 | 0.0025 |
| Year | 1 | 3.0181 | 20.655 | -28.329 | 0.0054 | 0.0115 |
| Org_S_ | 1 | 1.1505 | 18.787 | -32.973 | 0.0785 | 0.0982 |
| Treatment | 1 | 0.3634 | 18 | -35.07 | 0.3175 | 0.3243 |
| [Ca]_S_ | 1 | 1.4332 | 19.07 | -32.241 | 0.0504 | 0.0700 |
| Org_S_*Treatment | 1 | 1.5379 | 19.175 | -31.973 | 0.0430 | 0.0611 |

Table S2 (continued).

| [K]_S_*Treatment | 1 | 1.0694 | 18.706 | -33.185 | 0.0894 | 0.1091 |
| --- | --- | --- | --- | --- | --- | --- |
| Water-use efficiency (WUE), first order | Df | Sum of Sq | RSS | AIC | Pr(Chi) | *q* |
| <none> |  |  | 259.64 | 76.139 |  |  |
| Treatment | 1 | 39.098 | 298.74 | 79.048 | 0.0267 | 0.0399 |
| [Mg]_S_ | 1 | 17.197 | 276.84 | 76.383 | 0.1341 | 0.1457 |
| Water-use efficiency (WUE), second order | Df | Sum of Sq | RSS | AIC | Pr(Chi) | *q* |
| <none> |  |  | 259.64 | 76.139 |  |  |
| Treatment | 1 | 39.098 | 298.74 | 79.048 | 0.0267 | 0.0399 |
| [Mg]_S_ | 1 | 17.197 | 276.84 | 76.383 | 0.1341 | 0.1457 |
| Volume increment:leaf area ratio (Δ*V*:*A*_LT_), first order | Df | Sum of Sq | RSS | AIC | Pr(Chi) | *q* |
| <none> |  |  | 299020 | 437.11 |  |  |
| [K]_S_ | 1 | 80383 | 379403 | 446.77 | 0.0006 | 0.0020 |
| Year | 1 | 64589 | 363608 | 444.69 | 0.0020 | 0.0048 |
| [N]_S_ | 1 | 42161 | 341181 | 441.57 | 0.0110 | 0.0191 |
| [P]_S_ | 1 | 13008 | 312028 | 437.19 | 0.1486 | 0.1571 |
| Volume increment:leaf area ratio (Δ*V*:*A*_LT_), second order | Df | Sum of Sq | RSS | AIC | Pr(Chi) | *q* |
| <none> |  |  | 267292 | 435.61 |  |  |
| [K]_S_ | 1 | 81927 | 349218 | 446.71 | 0.0003 | 0.0010 |
| Year | 1 | 718 | 268010 | 433.74 | 0.7169 | 0.7229 |
| [N]_S_ | 1 | 12419 | 279711 | 435.83 | 0.1358 | 0.1462 |
| [P]_S_ | 1 | 25469 | 292761 | 438.07 | 0.0347 | 0.0509 |
| Year*[P]_S_ | 1 | 25479 | 292771 | 438.07 | 0.0347 | 0.0511 |
| Year*[N]_S_ | 1 | 12433 | 279725 | 435.84 | 0.1356 | 0.1466 |
| Single leaf area (*A*_Li_), first order | Df | Sum of Sq | RSS | AIC | Pr(Chi) | *q* |
| <none> |  |  | 13877 | 1481 |  |  |
| Year | 1 | 11377.9 | 25255 | 1730.5 | <0.0001 | <0.0001 |
| Treatment | 1 | 1337.8 | 15215 | 1517.7 | <0.0001 | <0.0001 |
| [N]_S_ | 1 | 476.3 | 14353 | 1493.2 | 0.0002 | 0.0007 |
| Org_S_ | 1 | 284 | 14161 | 1487.6 | 0.0035 | 0.0081 |
| [P]_S_ | 1 | 80 | 13957 | 1481.5 | 0.1203 | 0.1369 |
| Single leaf area (*A*_Li_), second order | Df | Sum of Sq | RSS | AIC | Pr(Chi) | *q* |
| <none> |  |  | 11890 | 1420.2 |  |  |
| Year | 1 | 1185.73 | 13076 | 1458.1 | <0.0001 | <0.0001 |
| Treatment | 1 | 879.93 | 12770 | 1448.2 | <0.0001 | <0.0001 |
| [N]_S_ | 1 | 323.09 | 12214 | 1429.4 | 0.0008 | 0.0023 |
| *Y* | 1 | 1052.85 | 12943 | 1453.8 | <0.0001 | <0.0001 |
| Year*Treatment | 1 | 877.29 | 12768 | 1448.1 | <0.0001 | <0.0001 |
| Treatment*[N]_S_ | 1 | 389.82 | 12280 | 1431.7 | 0.0002 | 0.0008 |
| Year**Y* | 1 | 1053.08 | 12944 | 1453.8 | <0.0001 | <0.0001 |
| Single leaf weight (*W*_Li_), first order | Df | Sum of Sq | RSS | AIC | Pr(Chi) | *q* |
| <none> |  |  | 1.2409 | -2434.3 |  |  |
| Year | 1 | 0.8316 | 2.0725 | -2220.8 | <0.0001 | <0.0001 |
| Treatment | 1 | 0.14272 | 1.3836 | -2390.5 | <0.0001 | <0.0001 |
| [N]_S_ | 1 | 0.05197 | 1.2929 | -2419 | <0.0001 | 0.0002 |
| Org_S_ | 1 | 0.03169 | 1.2726 | -2425.7 | 0.0011 | 0.0031 |
| [P]_S_ | 1 | 0.00893 | 1.2498 | -2433.2 | 0.0827 | 0.1019 |

Table S2 (continued).

| Single leaf weight (*W*_Li_), second order | Df | Sum of Sq | RSS | AIC | Pr(Chi) | *q* |
| --- | --- | --- | --- | --- | --- | --- |
| <none> |  |  | 1.0551 | -2492.4 |  |  |
| Year | 1 | 0.007283 | 1.0623 | -2491.5 | 0.0892 | 0.1093 |
| Treatment | 1 | 0.063433 | 1.1185 | -2469.9 | <0.0001 | <0.0001 |
| [N]_S_ | 1 | 0.00876 | 1.0638 | -2490.9 | 0.0624 | 0.0833 |
| *Y* | 1 | 0.047513 | 1.1026 | -2475.9 | <0.0001 | 0.0001 |
| pH_S_ | 1 | 0.008427 | 1.0635 | -2491.1 | 0.0676 | 0.0882 |
| Year*Treatment | 1 | 0.063297 | 1.1184 | -2469.9 | <0.0001 | <0.0001 |
| Year*[N]_S_ | 1 | 0.008769 | 1.0638 | -2490.9 | 0.0623 | 0.0836 |
| Treatment*[N]_S_ | 1 | 0.02849 | 1.0836 | -2483.2 | 0.0008 | 0.0024 |
| Year**Y* | 1 | 0.047526 | 1.1026 | -2475.9 | <0.0001 | 0.0001 |
| Year* pH_S_ | 1 | 0.00843 | 1.0635 | -2491.1 | 0.0675 | 0.0887 |
| Leaf mass per area (LMA), first order | Df | Sum of Sq | RSS | AIC | Pr(Chi) | *q* |
| <none> |  |  | 35062 | 1868.3 |  |  |
| Treatment | 1 | 2082.05 | 37144 | 1890.6 | <0.0001 | <0.0001 |
| [K]_S_ | 1 | 317.75 | 35379 | 1870.1 | 0.0516 | 0.0704 |
| Org_S_ | 1 | 472.05 | 35534 | 1872 | 0.0178 | 0.0291 |
| [Mg]_S_ | 1 | 274.77 | 35336 | 1869.6 | 0.0702 | 0.0907 |
| Leaf mass per area (LMA), second order | Df | Sum of Sq | RSS | AIC | Pr(Chi) | *q* |
| <none> |  |  | 33417 | 1854.2 |  |  |
| Treatment | 1 | 1984.46 | 35402 | 1876.4 | <0.0001 | <0.0001 |
| [K]_S_ | 1 | 2159.24 | 35577 | 1878.5 | <0.0001 | <0.0001 |
| Org_S_ | 1 | 1413.9 | 34831 | 1869.6 | <0.0001 | 0.0002 |
| [N]_S_ | 1 | 863.39 | 34281 | 1862.9 | 0.0011 | 0.0029 |
| *Y* | 1 | 310.78 | 33728 | 1856 | 0.0486 | 0.0684 |
| Treatment*Org_S_ | 1 | 1257.56 | 34675 | 1867.7 | 0.0001 | 0.0004 |
| Treatment*[K]_S_ | 1 | 514.32 | 33932 | 1858.6 | 0.0113 | 0.0195 |
| Foliar N concentration ([N]_L_), first order | Df | Sum of Sq | RSS | AIC | Pr(Chi) | *q* |
| <none> |  |  | 0.35179 | -91.346 |  |  |
| *D* | 1 | 0.268884 | 0.62068 | -79.72 | 0.0002 | 0.0008 |
| Org_S_ | 1 | 0.109267 | 0.46106 | -86.855 | 0.0108 | 0.0192 |
| [Mg]_S_ | 1 | 0.043497 | 0.39529 | -90.549 | 0.0944 | 0.1128 |
| *X* | 1 | 0.035646 | 0.38744 | -91.03 | 0.1280 | 0.1410 |
| Foliar N concentration ([N]_L_), second order | Df | Sum of Sq | RSS | AIC | Pr(Chi) | *q* |
| <none> |  |  | 0.35179 | -91.346 |  |  |
| *D* | 1 | 0.268884 | 0.62068 | -79.72 | 0.0002 | 0.0008 |
| Org_S_ | 1 | 0.109267 | 0.46106 | -86.855 | 0.0108 | 0.0192 |
| [Mg]_S_ | 1 | 0.043497 | 0.39529 | -90.549 | 0.0944 | 0.1128 |
| *X* | 1 | 0.035646 | 0.38744 | -91.03 | 0.1280 | 0.1410 |
| Foliar P concentration ([P]_L_), first order | Df | Sum of Sq | RSS | AIC | Pr(Chi) | *q* |
| <none> |  |  | 0.0039492 | -203.09 |  |  |
| *D* | 1 | 0.00251604 | 0.0064652 | -193.26 | 0.0006 | 0.0018 |
| Treatment | 1 | 0.00098187 | 0.0049311 | -199.77 | 0.0210 | 0.0330 |
| Foliar P concentration ([P]_L_), second order | Df | Sum of Sq | RSS | AIC | Pr(Chi) | *q* |
| <none> |  |  | 0.0039492 | -203.09 |  |  |
| *D* | 1 | 0.00251604 | 0.0064652 | -193.26 | 0.0006 | 0.0018 |
| Treatment | 1 | 0.00098187 | 0.0049311 | -199.77 | 0.0210 | 0.0330 |
| Foliar K concentration ([K]_L_), first order | Df | Sum of Sq | RSS | AIC | Pr(Chi) | *q* |
| <none> |  |  | 0.092292 | -123.46 |  |  |

Table S2 (continued).

| Year | 1 | 0.053016 | 0.145308 | -114.57 | 0.0010 | 0.0027 |
| --- | --- | --- | --- | --- | --- | --- |
| Treatment | 1 | 0.043344 | 0.135636 | -116.22 | 0.0024 | 0.0057 |
| [Ca]_S_ | 1 | 0.021836 | 0.114128 | -120.36 | 0.0240 | 0.0374 |
| pH_S_ | 1 | 0.010193 | 0.102485 | -122.95 | 0.1128 | 0.1315 |
| Foliar K concentration ([K]_L_), second order | Df | Sum of Sq | RSS | AIC | Pr(Chi) | *q* |
| <none> |  |  | 0.082433 | -124.17 |  |  |
| Year | 1 | 0.004064 | 0.086497 | -125.02 | 0.2825 | 0.2935 |
| Treatment | 1 | 0.043344 | 0.125777 | -116.03 | 0.0015 | 0.0038 |
| [Ca]_S_ | 1 | 0.009851 | 0.092284 | -123.46 | 0.0998 | 0.1180 |
| pH_S_ | 1 | 0.010193 | 0.092626 | -123.37 | 0.0944 | 0.1139 |
| Year* [Ca]_S_ | 1 | 0.009859 | 0.092292 | -123.46 | 0.0996 | 0.1185 |
| Foliar Ca concentration ([Ca]_L_), first order | Df | Sum of Sq | RSS | AIC | Pr(Chi) | *q* |
| <none> |  |  | 1.4257 | -59.761 |  |  |
| *D* | 1 | 0.36959 | 1.7953 | -56.229 | 0.0187 | 0.0301 |
| Treatment | 1 | 0.49264 | 1.9183 | -54.638 | 0.0076 | 0.0144 |
| *H* | 1 | 0.14999 | 1.5757 | -59.36 | 0.1213 | 0.1374 |
| Foliar Ca concentration ([Ca]_L_), second order | Df | Sum of Sq | RSS | AIC | Pr(Chi) | *q* |
| <none> |  |  | 1.3167 | -61.67 |  |  |
| *D* | 1 | 1.52084 | 2.8376 | -45.243 | <0.0001 | 0.0001 |
| Treatment | 1 | 0.42116 | 1.7379 | -57.009 | 0.0099 | 0.0178 |
| *D**Treatment | 1 | 0.25897 | 1.5757 | -59.36 | 0.0379 | 0.0549 |
| Foliar Mg concentration ([Mg]_L_), first order | Df | Sum of Sq | RSS | AIC | Pr(Chi) | *q* |
| <none> |  |  | 0.023248 | -154.55 |  |  |
| Year | 1 | 0.01636 | 0.039608 | -143.76 | 0.0003 | 0.0012 |
| pH_S_ | 1 | 0.056588 | 0.079836 | -126.94 | <0.0001 | <0.0001 |
| *D* | 1 | 0.009175 | 0.032423 | -148.57 | 0.0047 | 0.0103 |
| *X* | 1 | 0.028009 | 0.051257 | -137.57 | <0.0001 | 0.0001 |
| *Y* | 1 | 0.002229 | 0.025477 | -154.35 | 0.1383 | 0.1482 |
| Foliar Mg concentration ([Mg]_L_), second order | Df | Sum of Sq | RSS | AIC | Pr(Chi) | *q* |
| <none> |  |  | 0.012191 | -162.04 |  |  |
| Treatment | 1 | 0.0092508 | 0.021442 | -150.49 | 0.0002 | 0.0009 |
| Year | 1 | 0.0000083 | 0.0122 | -164.03 | 0.8981 | 0.9019 |
| pH_S_ | 1 | 0.0069256 | 0.019117 | -153.25 | 0.0010 | 0.0028 |
| Org_S_ | 1 | 0.0051395 | 0.017331 | -155.6 | 0.0037 | 0.0082 |
| [K]_S_ | 1 | 0.0045684 | 0.01676 | -156.4 | 0.0057 | 0.0121 |
| [Ca]_S_ | 1 | 0.0039616 | 0.016153 | -157.29 | 0.0094 | 0.0172 |
| [N]_S_ | 1 | 0.0032971 | 0.015488 | -158.3 | 0.0165 | 0.0276 |
| Treatment*Year | 1 | 0.0092042 | 0.021395 | -150.54 | 0.0002 | 0.0009 |
| Treatment*Org_S_ | 1 | 0.0021104 | 0.014302 | -160.21 | 0.0503 | 0.0703 |
| Foliar N:P ratio ([N:P]_L_), first order | Df | Sum of Sq | RSS | AIC | Pr(Chi) | *q* |
| <none> |  |  | 0.0015192 | -226.02 |  |  |
| Treatment | 1 | 0.0005451 | 0.0020643 | -220.66 | 0.0067 | 0.0132 |
| [N]_S_ | 1 | 0.00026103 | 0.0017802 | -224.22 | 0.0511 | 0.0702 |
| Foliar N:P ratio ([N:P]_L_), second order | Df | Sum of Sq | RSS | AIC | Pr(Chi) | *q* |
| <none> |  |  | 0.0015192 | -226.02 |  |  |
| Treatment | 1 | 0.0005451 | 0.0020643 | -220.66 | 0.0067 | 0.0132 |
| [N]_S_ | 1 | 0.00026103 | 0.0017802 | -224.22 | 0.0511 | 0.0702 |

Table S2 (continued).

| Foliar N:K ratio ([N:K]_L_), first order | Df | Sum of Sq | RSS | AIC | Pr(Chi) | *q* |
| --- | --- | --- | --- | --- | --- | --- |
| <none> |  |  | 0.00020397 | -270.21 |  |  |
| Treatment | 1 | 0.00006335 | 0.00026732 | -265.72 | 0.0108 | 0.0189 |
| *X* | 1 | 0.000021583 | 0.00022555 | -269.8 | 0.1203 | 0.1375 |
| [Ca]_S_ | 1 | 0.000054476 | 0.00025844 | -266.53 | 0.0172 | 0.0283 |
| [P]_S_ | 1 | 0.000021054 | 0.00022502 | -269.86 | 0.1247 | 0.1386 |
| Foliar N:K ratio ([N:K]_L_), second order | Df | Sum of Sq | RSS | AIC | Pr(Chi) | *q* |
| <none> |  |  | 0.00020397 | -270.21 |  |  |
| Treatment | 1 | 0.00006335 | 0.00026732 | -265.72 | 0.0108 | 0.0189 |
| *X* | 1 | 0.000021583 | 0.00022555 | -269.8 | 0.1203 | 0.1375 |
| [Ca]_S_ | 1 | 0.000054476 | 0.00025844 | -266.53 | 0.0172 | 0.0283 |
| [P]_S_ | 1 | 0.000021054 | 0.00022502 | -269.86 | 0.1247 | 0.1386 |
| Total content of N in foliage ([N]_LT_), first | Df | Sum of Sq | RSS | AIC | Pr(Chi) | *q* |
| order |  |  |  |  |  |  |
| <none> |  |  | 6.8852 | -21.9682 |  |  |
| Year | 1 | 7.4193 | 14.3045 | -6.4195 | <0.0001 | 0.0002 |
| Treatment | 1 | 2.4215 | 9.3068 | -16.7354 | 0.0072 | 0.0139 |
| [K]_S_ | 1 | 2.4914 | 9.3767 | -16.5559 | 0.0065 | 0.0131 |
| Total content of N in foliage ([N]_LT_), second order | Df | Sum of Sq | RSS | AIC | Pr(Chi) | *q* |
| <none> |  |  | 5.7955 | -24.1032 |  |  |
| Year | 1 | 7.0979 | 12.8934 | -6.9121 | <0.0001 | 0.0001 |
| Treatment | 1 | 1.0889 | 6.8844 | -21.971 | 0.0421 | 0.0602 |
| [K]_S_ | 1 | 2.4914 | 8.287 | -17.5209 | 0.0034 | 0.0079 |
| Year*Treatment | 1 | 1.0897 | 6.8852 | -21.9682 | 0.0420 | 0.0605 |
| Total content of P in foliage ([P]_LT_), first order | Df | Sum of Sq | RSS | AIC | Pr(Chi) | *q* |
| <none> |  |  | 0.062685 | -136.75 |  |  |
| Year | 1 | 0.058608 | 0.121293 | -122.9 | 0.0001 | 0.0003 |
| [Mg]_S_ | 1 | 0.021948 | 0.084632 | -131.54 | 0.0073 | 0.0139 |
| Total content of P in foliage ([P]_LT_), second order | Df | Sum of Sq | RSS | AIC | Pr(Chi) | *q* |
| <none> |  |  | 0.062685 | -136.75 |  |  |
| Year | 1 | 0.058608 | 0.121293 | -122.9 | 0.0001 | 0.0003 |
| [Mg]_S_ | 1 | 0.021948 | 0.084632 | -131.54 | 0.0073 | 0.0139 |
| Total content of K in foliage ([K]_LT_), first order | Df | Sum of Sq | RSS | AIC | Pr(Chi) | *q* |
| <none> |  |  | 0.35794 | -92.931 |  |  |
| Year | 1 | 0.71795 | 1.07589 | -68.518 | <0.0001 | <0.0001 |
| [K]_S_ | 1 | 0.18098 | 0.53892 | -85.11 | 0.0017 | 0.0043 |
| Treatment | 1 | 0.05085 | 0.40879 | -91.743 | 0.0742 | 0.0943 |
| Total content of K in foliage ([K]_LT_), second order | Df | Sum of Sq | RSS | AIC | Pr(Chi) | *q* |
| <none> |  |  | 0.35794 | -92.931 |  |  |
| Year | 1 | 0.71795 | 1.07589 | -68.518 | <0.0001 | <0.0001 |
| [K]_S_ | 1 | 0.18098 | 0.53892 | -85.11 | 0.0017 | 0.0043 |
| Treatment | 1 | 0.05085 | 0.40879 | -91.743 | 0.0742 | 0.0943 |

Table S2 (continued).

| Total content of Ca in foliage ([Ca]_LT_), first order | Df | Sum of Sq | RSS | AIC | Pr(Chi) | *q* |
| --- | --- | --- | --- | --- | --- | --- |
| <none> |  |  | 5.4847 | -27.426 |  |  |
| Year | 1 | 7.2831 | 12.7678 | -9.147 | <0.0001 | <0.0001 |
| [N]_S_ | 1 | 2.4028 | 7.8875 | -20.706 | 0.0031 | 0.0074 |
| Org_S_ | 1 | 1.3669 | 6.8516 | -24.086 | 0.0208 | 0.0334 |
| Total content of Ca in foliage ([Ca]_LT_), second order | Df | Sum of Sq | RSS | AIC | Pr(Chi) | *q* |
| <none> |  |  | 4.4916 | -28.22 |  |  |
| Year | 1 | 0.06102 | 4.5527 | -29.896 | 0.5693 | 0.5765 |
| [N]_S_ | 1 | 0.51518 | 5.0068 | -27.614 | 0.1065 | 0.1253 |
| Org_S_ | 1 | 1.72538 | 6.217 | -22.418 | 0.0052 | 0.0112 |
| Year*[N]_S_ | 1 | 0.51479 | 5.0064 | -27.616 | 0.1066 | 0.1249 |
| [N]_S_*Org_S_ | 1 | 0.47827 | 4.9699 | -27.792 | 0.1192 | 0.1376 |
| Total content of Mg in foliage ([Mg]_LT_), first order | Df | Sum of Sq | RSS | AIC | Pr(Chi) | *q* |
| <none> |  |  | 0.14203 | -117.11 |  |  |
| Year | 1 | 0.073815 | 0.21585 | -109.07 | 0.0015 | 0.0040 |
| [K]_S_ | 1 | 0.029068 | 0.1711 | -114.65 | 0.0345 | 0.0512 |
| Total content of Mg in foliage ([Mg]_LT_), second order | Df | Sum of Sq | RSS | AIC | Pr(Chi) | *q* |
| <none> |  |  | 0.082749 | -122.08 |  |  |
| *H* | 1 | 0.0036551 | 0.086404 | -123.04 | 0.3084 | 0.3164 |
| [P]_S_ | 1 | 0.0129384 | 0.095687 | -120.59 | 0.0619 | 0.0835 |
| [Ca]_S_ | 1 | 0.0038252 | 0.086574 | -123 | 0.2977 | 0.3067 |
| pH_S_ | 1 | 0.0078482 | 0.090597 | -121.91 | 0.1403 | 0.1497 |
| *H**[P]_S_ | 1 | 0.0224255 | 0.105175 | -118.33 | 0.0164 | 0.0277 |
| *H**[Ca]_S_ | 1 | 0.0104481 | 0.093197 | -121.23 | 0.0912 | 0.1106 |
